# Functionalized siRNA-chitosan nanoformulations promote triple-negative breast cancer cell death via blocking the miRNA-21/AKT/ERK signaling axis: in-silico and in vitro studies

**DOI:** 10.1007/s00210-024-03068-w

**Published:** 2024-04-09

**Authors:** Shaymaa A. Abdulmalek, Abdulrahman M. Saleh, Yasmin R. Shahin, Eman Fawzy El Azab

**Affiliations:** 1 Department of Biochemistry, Faculty of Science, Alexandria University, Alexandria, 21511 Egypt; 2 Department of Pharmaceutical Chemistry, Faculty of Pharmacy, Cairo University, Kasr El‑Aini Street, Cairo, 11562 Egypt; 3 Aweash El-Hagar Family Medicine Center, Epidemiological Surveillance Unit, MOHP, Mansoura, 35711 Egypt; 4 Department of Clinical Laboratory Sciences, College of Applied Medical Sciences at Al‐Qurayyat, Jouf University, Al‐Qurayyat, 77454 Saudi Arabia

**Keywords:** Breast cancer, EGFR overexpression, miRNA-21, Apoptosis, Targeted therapy, Molecular docking, AKT, siRNA

## Abstract

Oncogenic microRNA (miRNA), especially miRNA-21 upregulation in triple-negative breast cancer (TNBC), suggests a new class of therapeutic targets. In this study, we aimed to create GE11 peptide-conjugated small interfering RNA-loaded chitosan nanoparticles (GE11-siRNA-CSNPs) for the targeting of EGFR overexpressed TNBC and selectively inhibit miRNA-21 expression. A variety of in-silico and in vitro cellular and molecular studies were conducted to investigate the binding affinities of specific targets used as well as the anticancer efficacies and mechanisms of GE11-siRNA-CSNPs in TNBC cells. An in-silico assessment reveals a distinct binding affinity of miRNA-21 with siRNA as well as between the extracellular domain of EGFR and synthesized peptides. Notably, the in vitro results showed that GE11-siRNA-CSNPs were revealed to have better cytotoxicity against TNBC cells. It significantly inhibits miRNA-21 expression, cell migration, and colony formation. The results also indicated that GE11-siRNA-CSNPs impeded cell cycle progression. It induces cell death by reducing the expression of the antiapoptotic gene Bcl-2 and increasing the expression of the proapoptotic genes Bax, Caspase 3, and Caspase 9. Additionally, the docking analysis and immunoblot investigations verified that GE1-siRNA-CSNPs, which specifically target TNBC cells and suppress miRNA-21, can prevent the effects of miRNA-21 on the proliferation of TNBC cells via controlling EGFR and subsequently inhibiting the PI3K/AKT and ERK1/2 signaling axis. The GE11-siRNA-CSNPs design, which specifically targets TNBC cells, offers a novel approach for the treatment of breast cancer with improved effectiveness. This study suggests that GE11-siRNA-CSNPs could be a promising candidate for further assessment as an additional strategy in the treatment of TNBC.

**Graphical Abstract:**

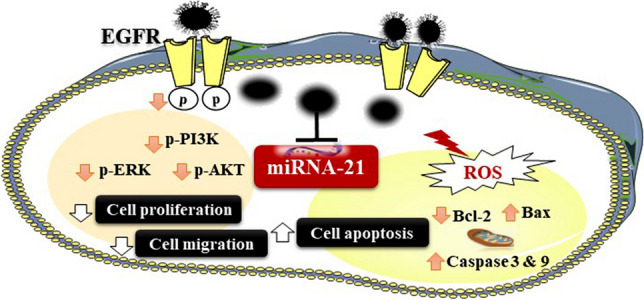

## Introduction

Breast cancer is a common female malignant tumor that poses a major threat to women’s health. In 2020, the World Health Organization reported that 2.3 million women worldwide were diagnosed with breast cancer and 685,000 women lost their lives. Additionally, 43,170 women and 530 men were expected to die from the breast cancer in 2023 (Siegel et al. 2023). Around 15% of breast tumors have triple-negative breast cancers (TNBC), which are often distinguished by a poor prognosis and high rates of proliferation and metastasis (Dai et al. 2017; Giovannelli et al. 2019). It was found that epidermal growth factor receptor (EGFR) was overexpressed TNBC, which was inversely connected with clinical prognosis, and that it was intimately associated with the proliferation, invasion, and vascular development of cancer (Pal et al. 2024). TNBC is a prominent cause of cancer deaths in women due to its aggressive nature and a lack of effective therapy methods (Berger et al. 2021; Akter et al. 2023). Studies have shown that TNBC formation and occurrence involved abnormally active mitogen-activated protein kinase/extracellular signal-regulated kinases (MAPK/ERK), which may provide TNBC the capacity to multiply and withstand apoptosis (Kim et al. 2016; Ryu and Sohn 2021; Pradhan et al. 2023; Yang et al. 2023a, b).

It has been reported that microRNAs (miRNAs) function as negative gene regulators, inhibiting translation and degrading mRNA to repress the expression of the target gene (Danai et al. 2023; Fathi et al. 2023). In practically all human malignancies over the past 10 years, dysregulation of miRNAs has been connected to tumor initiation, development, and metastasis (Yanaihara et al. 2006). miRNAs have been generally referred to as “oncomiRs” because they have the potential to act as both tumor suppressors and oncogenes (Esquela-Kerscher and Slack 2006). The majority of cancer cell lines including breast cancer are affected by miRNA-21, making it typical among the oncomiRs discovered so far (Deng et al. 2017). miRNA-21 is a positive feedback regulator of the MAPK/ERK1/2 pathways. Its own expression is promoted by activation of ERK1/2, whose activity is subsequently increased by suppressing ERK/MAPK negative regulators. miRNA-21 promotes cancer cell proliferation, survival, and migration by targeting phosphatase and tensin homolog (PTEN), the major negative regulator of the PI3K pathway, resulting in increased phosphoinositide 3-kinases (PI3K) activity (Yang et al. 2011). One of the most important techniques in cancer treatment is targeting the inhibition of oncogene expression. Knockdown techniques for genes involved in cancer etiology have been tried and failed to entirely silence the gene (Watts and Corey 2012). It has been found that RNA interference (RNAi) is a revolutionary technique for modifying gene expressions. The small interfering RNA (siRNA) was loaded into the RNA-induced silencing complex (RISC) effector complex to unwind, where single-stranded RNA hybridizes with mRNA targets, resulting in gene silencing and protein down-regulation (Chen et al. 2018; Tufail and Wu 2023; Cömez and Akbuğa 2023).

When comparing siRNA with conventional pharmaceutical therapies, there are certain advantages. Hence, the identification of innovative and advanced molecular targets would enhance the available treatment choices for breast cancer. Indeed, transporting siRNA can be difficult due to the instability of unbound siRNA and its ability to pass across cell membranes. So, numerous attempts have been made, like using particular genes linked to tumor growth and development, using an effective siRNA delivery strategy, and functionalizing the carrier for siRNA targetability (Ashique et al. 2022‏). Nanoparticles like chitosan nanoparticles (CSNPs) are thought to be biodegradable, biocompatible, and low-toxic, and are therefore frequently used as siRNA delivery systems (Babu et al. 2017). siRNAs can be protected against biological degradation and rapid clearance when delivered via biodegradable and biocompatible non-viral vectors (Kanasty et al. 2013). These nano-formulations increase the oral bioavailability of siRNA by making the molecule more soluble in serum and less susceptible to degradation, and the nano-encapsulation of siRNA modifies its pharmacokinetic properties by shielding them from degradation in serum and elimination by the kidneys and liver. In addition, the resistance of cancer cells to siRNAs is reduced and cellular internalization and intracellular drug release are enhanced by stimuli-mediated nano-therapeutics (Opriș et al. 2024). An ideal nanocarrier should be able to transmit siRNAs in a manner that is biocompatible, biodegradable, safe, and non-immunogenic; these are the most important goals of an ideal nanocarrier (Bayda et al. 2018). Because they interfere with the late translational stage of gene expression, siRNA-based therapies are non-teratogenic and mutagenic, and they are particularly effective because they decrease targeted genes selectively and preferentially (Mishra et al. 2017). To knock down any gene in the intended tumor cells with low immunogenicity and off-target effects, siRNA-based therapies can be easily designed and tweaked (Parvani and Jackson 2017; Hattab and Bakhtiar 2020; Chaudhuri et al. 2022).

Considering the critical role of miRNA-21 inhibition in TNBC cells as a non-invasive method, this study aims to examine how GE11-siRNA-CSNPs can selectively target and silence miRNA-21 in TNBC cells. We hypothesized that the targeting of EGFR could be a therapeutic strategy for treating breast cancer. We contend that the proliferation and invasiveness of TNBC may be reduced using this synthetic targeted treatment. To assess the potentiality of prepared nanoparticles, several in vitro experiments against TNBC cells were investigated, also we examined the expression of EGFR and the downstream proteins. Furthermore, we investigated the computational interaction of siRNA with miRNA-21, as well as the binding of the extracellular domain of EGFR with generated peptides. Additionally, we analyzed the binding affinity between miRNA-21 and AKT protein. GE11-siRNA-CSNPs that were prepared demonstrated an augmented anticancer effect by triggering cancer cells death.

## Materials and methods

### Chemicals

MDA-MB-231 (RRID: CVCL_0062) and MCF7 (RRID: CVCL_0031) cells were purchased from American Type Culture Collection (ATCC, Manassas, Virginia, USA). A kit for apoptosis detection using annexin V and fluorescein isothiocyanate (FITC) was bought from Cell signaling technology (Beverly, MA, USA). DSPE-PEG (2000) Maleimide, chitosan (purity 98%), RNase-free eppendorf tubes, acetic acid, 5-diphenyltetrazolium bromide (MTT), and protein assay kit were purchased from Sigma-Aldrich (St. Louis, MO, USA). The following items were purchased from Gibco, Thermo Fisher Scientific, Inc. (Waltham, MA, USA): Dulbecco’s modified Eagle medium (DMEM), 0.25% trypsin–EDTA, penicillin–streptomycin, fetal bovine serum, ROS kit, and GE11 polypeptide. QIAzol Lysis Reagent (#79306) (QIAGEN, Hilden, Germany), miRNeasy Mini kit (QIAGEN, Inc, 217004), miScript® II RT Kit (QIAGEN, Inc, 21861), miScript Primer Assay (forward primer) (QIAGEN, 218300, MS00009079), miScript SYBR Green PCR Kit (QIAGEN, 218073), SensiFAST cDNA Synthesis Kit (BIO-65054), and SensiFAST SYBR Green No-ROX Kit (BIO-98005) (Meridian Bioscience, Cincinnati, OH, USA). RIPA lysis buffer, p-EGFR (3777), p-ERK (4377), p-Akt (Ser473) (4060), p-PI3K p85 (17,366), β-actin (4970), anti-rabbit IgG (7054), and U0126 (ERK inhibitor) were purchased from Cell Signaling Technology (Beverly, MA, USA). Alkaline phosphatase chromogen (BCIP/TNBT) (ab7413) was purchased from Abcam (Cambridge, UK).

### Method of in-silico simulation studies

#### Molecular docking siRNA against miRNA-21

The computational (in-silico) technique has been widely used as an efficient tool for virtual biological screening. The 3D crystal structure of wild-type pre-miRNA-21 apical loop (Oncogenic miRNA-21 precursor) was downloaded from the Protein Data Bank, http://www.rcsb.org/pdb (PDB ID: 5UZT) and assumed for this study. At first, the crystalized water molecules were excluded from the downloaded crystal structure. Molecular preparation was done to add the hydrogen atoms. Energy minimization was performed by applying CHARMM force field. Then, the hydrogen atoms were hidden to make the areas of interaction clearer. The 3D structure of the tested siRNA/miRNA-21 inhibitor was generated from the next sequence (5′-UCAACAUCAGUCUGAUAAGCUA-3′) by using RNA-Composer server. Then, protonation and energy of inhibitor was minimized by applying CHARMM force field. The docking process was done using the Ligand scout 2.0 software (based on Autodock vina) using blind docking technique; about twenty poses were predicted and the best orientation was chosen, then the 3D binding modes were generated by BIOVIA Discovery Studio Visualizer (Tessaro and Scapozza 2020).

#### GE11 peptides de novo prediction

The 3D structure of the target peptide is not present in the protein data bank (PDB), so we used artificial intelligence (AI) to do the de novo prediction of 3D structure from the GE11 peptide sequence (peptide sequence: YHWYGYTPQWVI). 3D structures of peptides were prepared by the I-TASSER server and visualized by Biovia Discovery Studio 2019 software.

### Structure validation of tested peptide

The best conformation (decoys) of 3D peptide structures selected by doing the homology modeling using the templet protein has structure similarity to detect the best configuration of alpha helix or beta sheets in predicted peptides. The I-TASSER modeling process commences with the structure templates that have been found by LOMETS from the PDB database. LOMETS is a metaserver threading method that consists of many threading programs. Each threading program has the ability to produce tens of thousands of template alignments. I-TASSER exclusively uses the templates with the utmost significance in the threading alignments, as determined by the Z-score. After the generation of the best conformation, I-TASSER algorithms selected the best one with a suitable C-score equal (C-score =  − 0.48) for molecular modeling.

### Protein-GE11 molecular docking

Protein–protein docking was done to better understand the inhibitory functions of the tested peptide against the extracellular domain of EGFR TK. MOE software was used to perform the docking technique. First water molecules have been removed from the complex. Then, crystallographic disorders and unfilled valence atoms were corrected using protein reports, utility, and clean protein options. EGFR TK and tested peptide-energy were minimized by applying MMFF94 force fields. The docking process was done using the Ligand Scout 2.0 software (based on Autodock vina) using the blind docking technique, about twenty poses were predicted, and the best orientation was chosen, then the 3D and 2D binding modes were generated by the BIOVIA Discovery Studio Visualizer (Vakser 2014).

### Preparation of targeted GE11-siRNA-CSNPs and non-targeted siRNA-CSNPs

First, 150 mg of chitosan was dissolved in 5 mL of acetic acid aqueous solution, and it was agitated for 20 min. The solution was mixed with 15 mL of distilled water, stirred (LCD Digital Hotplate Magnetic Stirrer, Laguna Hills, CA, USA), and left overnight before being vacuum-dried and kept at room temperature. After that, 50 mg of maleimide-poly-ethylene glycol-*N*-hydroxysuccinimide was combined with 50 mL of CSNPs solution for conjugation. The reaction was started and allowed to sit for three hours at room temperature and then stirred overnight. The solution was dialyzed using Milli-Q water, with half of the mixture being employed for an EGFR-binding peptide (Schäfer et al. 2011). In summary, PEG-CSNPs solution and GE11 were combined, and the mixture was constantly stirred for 4 h and allowed to react overnight at 4 ℃ under inert condition, so that the maleimide group and the peptide’s cysteine group could react to obtain GE11-targeted CSNPs. Lastly, siRNA was loaded using the produced nanoformulations. The mixture of 1 mL CSNPs or GE11-targeted CSNPs and 20 μL of siRNA (equivalent to 2.5 μg) was vortexed for 30 s at 3000 rpm and then allowed to sit at room temperature for 2 h in order to create non-targeted siRNA-CSNPs and GE11-siRNA-CSNPs (Malhotra et al. 2013; Joshi et al. 2021). Then the formation of GE11-siRNA-CSNPs complex was examined using gel electrophoresis. An agarose gel was loaded with 20 μL of the GE11-siRNA-CSNPs suspension using a loading dye dilution of 5:1. For 30 min, electrophoresis was run at 80 mA and a ChemiDoc Imaging Systems (Bio-Rad, Hercules, CA, USA) was utilized to visualize the siRNA bands, with free siRNA serving as the control.

### Characterization of prepared nanoparticles

The size, charge, shape, and encapsulation efficiency of the produced nanoparticles were evaluated. Dynamic light scattering (DLS) (Malvern Instruments, Malvern, UK) was used to calculate the polydispersive index (PDI), zeta potentials, and mean particle size of nanoparticles. The transmission electron microscope (TEM) (JEOL JEM-1400Flash, Tokyo, Japan) was used to study the morphological traits. Then, the formula was used to determine the effectiveness of siRNA encapsulation in the supernatant of nanoparticle formulation at 260 nm wavelength using UV-spectrophotometer (U.V-1601; Shimadzu, Japan) after centrifugation (Optima L-100 XP Ultracentrifuge with a rotor NV 70.1, Beckman-Coulter, USA) (18,000 rpm, 20 min, 4 ℃): Encapsulation Efficiency (%) = ((total amount of siRNA − free siRNA)/ (total amount of siRNA)) × 100.

### In vitro release profile and stability of synthesized nanoparticles

In RNase-free Eppendorf tubes, the GE11-siRNA-CSNPs are dissolved in 4 mL of PBS buffer at pH 7.4 or 6 while being shaken at 100 rpm and 37 ℃ (MS MP8 Wise Stir Wertheim, Germany). The entire material was collected by centrifugation at 14,000 rpm for 30 min at specified intervals of 0, 0.5, 1, 2, 4, 6, 8, 12, 20, 20, 24, 36, 48, and 72 h. Using a UV spectrophotometer, the amount of siRNA in supernatant at each time point was measured at 260 nm and substituted with the same volume of fresh buffer solution (Katas et al. 2013). In addition, gel electrophoresis was utilized to evaluate the serum stability of EGFR targeted siRNA-CSNPs. Briefly, a total of 200 μL of GE11-siRNA-CSNPs (containing 5 μg of siRNA) and 100 μL of FBS were combined and gently agitated at room temperature. As a control, naked siRNA received the same treatment. Subsequently, a precise volume of the samples (20 μL) was collected and stored at – 20 ℃, and their stability was assessed by subjecting them to analysis using a 4% agarose gel electrophoresis using 0.5 × TBE buffer, for 30 min at 4, 8, 16, 24, 36, and 48 h following incubation. After that, siRNA bands were visualized (ChemiDoc Imaging Systems, Bio-Rad, Hercules, CA, USA) (Salehi Khesht et al. 2021).

### Cell culture conditions

MDA-MB-231 and MCF-7 were grown to confluence at 37 ℃ in 5% CO_2_. High-glucose Dulbecco modified Eagle’s medium (DMEM) was used to sustain MDA-MB-231 and MCF-7. It also contained 10% heat-inactivated fetal bovine serum (FBS) and 1% penicillin/streptomycin for antibiotics. The freezing media consisted of dimethyl sulfoxide (DMSO) diluted with FBS at a concentration of 10%. Cells were sub-cultured when they had achieved 80% confluence by aspirating the media, rinsing the cells in PBS, and then adding 10X trypsin/EDTA for 2 min. FBS was added to full media after the cells were separated from the flask to inactivate the trypsin. The cells were then moved and divided into several flasks for more study. Every 3 days, the media was replaced, and the cells were divided once a week.

### Assessment of EGFR expression by Western blotting

About 1 × 10^5^ MDA-MB-231 or MCF7 cells were added to 6-well growth plates for Western blotting. Before being lysed with RIPA cell lysis solution and a protease inhibitor cocktail, the medium was removed, and the cells were washed twice with ice-cold PBS. Following the removal of the lysates from the plates, centrifugation was performed at 12,000 g for 5 min at 4 ℃. The BCA protein assay technique was used to measure protein concentrations. Briefly, 2X loading buffer (130 mM Tris–HCl, pH 8.0, 30% (v/v) Glycerol, 4.6% (w/v) SDS, 0.02% Bromophenol blue, and 2% DTT) was mixed with 50 µg of protein lysates from each sample, boiled for 5 min, and then chilled at 4 °C. Samples were run at 120 v while being separated on a 12% SDS-PAGE mini-gel (Mini-PROTEAN System, Bio-Rad, Hercules, CA, USA). Proteins were transferred to a nitrocellulose membrane (Trans-Blot® Turbo™ Transfer System, Bio-Rad, Hercules, CA, USA). The membrane was washed three times in TBST (50 mM Tris, pH 7.5, 150 mM NaCl, 0.05% Tween-20), blocked for an hour at room temperature with TBST that contains 5% nonfat dry milk, and then incubated overnight with primary antibodies (EGFR and β-actin) diluted in TBST. The membrane was treated with a secondary antibody for 1 h at room temperature following three TBST washes. Bands were detected using an alkaline phosphatase solution (Burnette 1981).

### Cytotoxicity evaluation using viability assay

The MDA-MB-231 cells were grown on DMEM medium supplemented with 10% FBS and 50 µg/mL gentamycin, at a concentration of 2 × 10^6^ cell/well in Corning® 96-well tissue culture plates, for the cytotoxicity assay. The cells were then incubated for 24 h at 37 ℃ in a humidified atmosphere with 5% CO_2_. To achieve six different concentrations for each formula, 100 µL of the nanoparticle solution was added onto 96-well plates (three replicates). These concentrations were 300, 100, 30, 10, 3, 1, 0.3, 0.1, and 0.03 μg/mL. MTT prepared in medium was added and incubated for 4 h after the media had been removed after 48 h of incubation. The final concentration of MTT was 0.5 mg/mL. Following the removal of the solution, DMSO was added, and it was incubated at 37 ℃ for 10 min. Cells that were not treated acted as a negative control. The absorbance of formazan solutions was measured at 570 nm using multi-well plate reader (BMGLABTECH®FLUOStar Omega, Germany). Using the GraphPad Prism program, the IC_50_ was determined (Mosmann 1983).

### miRNA-21 expression detection by RT-PCR

For evaluating the feasibility of miRNA-21 inhibition by GE11-siRNA-CSNPs, RT-PCR of miRNAs was carried out. Total RNA including miRNAs was extracted from MDA-MB-231 cells using miRNeasy Mini kit. The concentration and purity of total RNA was quantified by Thermo Scientific NanoDrop 1000 Spectrophotometer (Thermo Scientific, Waltham, MA, USA) and expressed as µg/µL. Also, a reverse transcription reaction was then performed using miScript® II RT Kit. For detection of mature miRNA, cDNA prepared in a reverse transcription reaction using miScript HiSpec Buffer serves as the template for RT-PCR analysis using an miRNA21-specific miScript primer assay (forward primer) and the miScript SYBR Green PCR Kit, which contains the miScript universal primer (reverse primer) and QuantiTect SYBR Green PCR Master Mix (CFX96 Touch Real-Time PCR Detection System, Bio-Rad, Hercules, CA, USA). Finally, the relative miRNA-21 expression was measured based on 2^−ΔΔCT^ method.

### Colony formation assay

As previously mentioned, colony development tests were conducted. Then, 500 MDA-MB-231 cells were cultured into 6-well plates, and the cells were allowed to develop overnight. The cells were subsequently exposed to GE11-siRNA-CSNPs for 2 h. The cells were washed in PBS after the drug-containing media was removed, and then they were cultivated for a further 10 days in a complete medium to form colonies. The cells were then fixed with 2% paraformaldehyde and washed twice with PBS. After that, each well of 6-well plates containing fixed colonies received 1 mL of (1%, 25 mM) crystal violet and was incubated at room temperature for 30 min. After the dye was removed, the wells were washed three times with dH_2_O and then twice with PBS. In five magnification fields chosen at random, cell colonies were calculated using ImageJ 1.47v software (http://imagej.nih.gov/ij) (Franken et al. 2006).

### Wound healing assay

In 6-well culture plates, MDA-MB-231 cells were cultured at a density of 2 × 10^5^ cells/well and expanded until 100% confluence. In a sterile setting, a 200 µL pipette tip was used to forcefully press against the tissue culture plate’s top, create a vertical wound through the confluent cell monolayer, and wash each well with PBS to remove non-adherent cells. The medium and cell debris were thoroughly aspirated. Then, enough culture media was added to the well wall to cover the bottom of the well and stop further cell detachment. The first image was then captured. At 37 ℃ and 5% CO_2_, the tissue culture plate was incubated. GE11-siRNA-CSNPs were applied to the cells at concentrations (0, 6, 12, 30 µg/mL), and they were subsequently incubated at the indicated time intervals. Under a microscope, the border of the center cell-free zone was verified. Using a microscope set at ×100 magnification, images were taken at 0, 24, 48, 72, and 96 h, and the wound area was calculated using ImageJ 1.47v software (http://imagej.nih.gov/ij) (Justus et al. 2014). Using MII ImageView 3.7v software, wound width was calculated as the average distance between the edges of the scratches.

### Cell cycle analysis

The MDA-MB-231 cell line was subjected to GE11-siRNA-CSNPs at concentrations of 12 µg/mL or 30 µg/mL or control media. The treatments were administered for a duration of 48 h. Following administration of treatment, trypsinization was used to gather cells (10^5^). Ice-cold PBS (pH 7.4) was then used twice to wash the cells. After being resuspended in two milliliters of 60% ice-cold ethanol, the cells were fixed for 1 h at 4 ℃. After repeatedly washing the fixed cells in PBS (pH 7.4), they were again suspended in 1 mL of PBS containing 10 µg/mL propidium iodide (PI) and 50 µg/mL RNAase A. Following a 20-min dark incubation period at 37 ℃, cells were subjected to flow cytometry analysis utilizing a FL2 (λex/em 535/617 nm) signal detector (ACEA NovocyteTM flowcytometer, ACEA Biosciences Inc., San Diego, CA, USA) to determine the DNA content of the cells. We collected 12,000 events for every sample. The ACEA NovoExpressTM program (ACEA Biosciences Inc., San Diego, CA, USA) was used to compute cell cycle distribution.

### Measurement of intracellular ROS

MDA-MB-231 cells were isolated, centrifuged twice, and suspended in PBS. The cells were then stained using the ROS detection kit as directed by the manufacturer at 37 ℃ for 30 min. After that, the cells were incubated with GE11-siRNA-CSNPs and non-targeted siRNA-CSNPs (100 µL/well) for varying lengths of time at 37 ℃. Using a microplate reader to determine intracellular ROS levels, cell fluorescence intensity was evaluated (excitation and emission wavelength set as 485/535 nm) (Bass et al. 1983).

### Gene expression of apoptotic markers by quantitative real-time-polymerase chain reaction

About 1 × 10^5^ cells/mL of MDA-MB-231 cells were cultured into 25 cm^2^ TPP-Swiss flasks. Different concentrations of prepared GE11-siRNA-CSNPs were applied to cancer cells for 48 h while also considering an untreated flask. The afflicted cells were separated from the remaining cells, which were then trypsinized and centrifuged at 4 ℃. PBS was used to rinse the pelted cells before transferring them to Eppendorf tubes. Using QIAzol Lysis reagent and following the manufacturer’s instructions, total RNA was extracted. Following the manufacturer’s instructions, cDNA products were produced from RNA using the SensiFAST cDNA synthesis kit. A thermal cycler was used to operate the reaction mixture (CFX96 Touch Real-Time PCR Detection System, Bio-Rad, Hercules, CA, USA). The RNA concentration was determined from the threshold cycle (Ct) values. The mRNA expression levels were calculated relative to the GAPDH gene’s mRNA levels using 2^−ΔΔCT^ method (Livak and Schmittgen 2001). Primer sequence: BCL2 (NM_000657.3) F: 5ʹ-ATGTGTGTGGAGACCGTCAA-3ʹ and R: 5ʹ-GCCGTACAGTTCCACAAAGGG-3ʹ. The Bax (NM_138763.4) F: 5ʹ-ATGTTTTCTGACGGCAACTTC-3ʹ and R: 5ʹ-AGTCCAATGTCCAGCCCAT-3ʹ. Caspase-9 (NM_001229.5) F: 5ʹ-CATTTCATGGTG-GAGGTGAAG-3ʹ and R: 5ʹ-GGGAACTG-CAGGTGGCTG-3ʹ. Caspase-3 (NM_004346.4) F: 5ʹ-TGTTTGTGTGCTTCTGAGCC-3ʹ and R: 5ʹ-CACGCCATGTCATCATCAAC-3ʹ. GAPDH (NM_001256799.3) F: 5ʹ-GGCACAGTCAAGGCTGAGAATG-3ʹ and 5′ R: 5ʹ- ATGGTGGTGAAGACGCCAGTA-3ʹ.

### Western blotting

After 48 h of treatment with different concentrations of GE11-siRNA-CSNPs, levels of the proteins BCL2, Bax, p-AKT, p-PI3K p58, and p-ERK1/2 was measured by Western blot analysis. As already described above, following the primary antibodies incubation, alkaline phosphatase chromogen was used to produce the membrane (Burnette 1981). U0126 treatment, TNBC cells were divided into four groups: control TNBC, U0126 (ERK1/2 inhibitor) (10 µM), GE11-siRNA-CSNPs (10 μM), and U0126 + GE11-siRNA-CSNPs treated cells. After 24 h, the cells were collected for western blot assay and detection of p-ERK1/2.

### Molecular docking of miRNA-21 and AKT

The blind docking technique was used to dock miRNA-21 to the target site of AKT. This was done to evaluate the potential affinity and inhibitory activity of miRNA-21 toward this target protein. Applying the above-described molecular docking method for the interaction between EGFR and GE11 peptide.

### Hypoxic cytotoxicity assay

A hypoxic environment with 0.5% O_2_ and 5% CO_2_ was produced with the use of hypoxic chambers (Anaero Pack, Mitsubishi Gas Chemical Co., Tokyo, Japan) according to the manufacturer’s recommendations, and the SRB assay was used to measure cell viability. In 96-well plates, aliquots of 100 μL cell suspension (5 × 10^3^ cells) were incubated for 24 h in full medium. Another aliquot of 100 μL medium containing medications at different concentrations (0.01, 0.1, 1, 10, and 100 μg/mL) were added to treat the cells. After drug treatment, cells were fixed by replacing medium with 150 μL of 10% TCA and incubated at 4 ℃ for 1 h. After removing the TCA solution, the cells underwent five rounds of distilled water washing. Once added, aliquots of 70 μL SRB solution (0.4% w/v) were incubated for 10 min at room temperature in a dark area. After three 1% acetic acid washes, the plates were left to air dry for the entire night. After that, 150 μL of TRIS (10 mM) was added to dissolve the protein-bound SRB stain, and an Infinite F50 microplate reader (TECAN, Männedorf, Switzerland) was used to measure the absorbance at 540 nm (Skehan et al. 1990).

### Statistical analyses

Statistical analyses were performed with GraphPad Prism v8 (GraphPad Software Inc.), and SPSS Predictive Analytics Software (IBM, Version 26). Multiple comparisons were made using one-way ANOVA with Tukey’s post-hoc test, and differences with a significance level of *p* < 0.01 were considered to be statistically significant.

## Results

### Binding mode of interaction of siRNA with miRNA-21

**Fig. 1:**
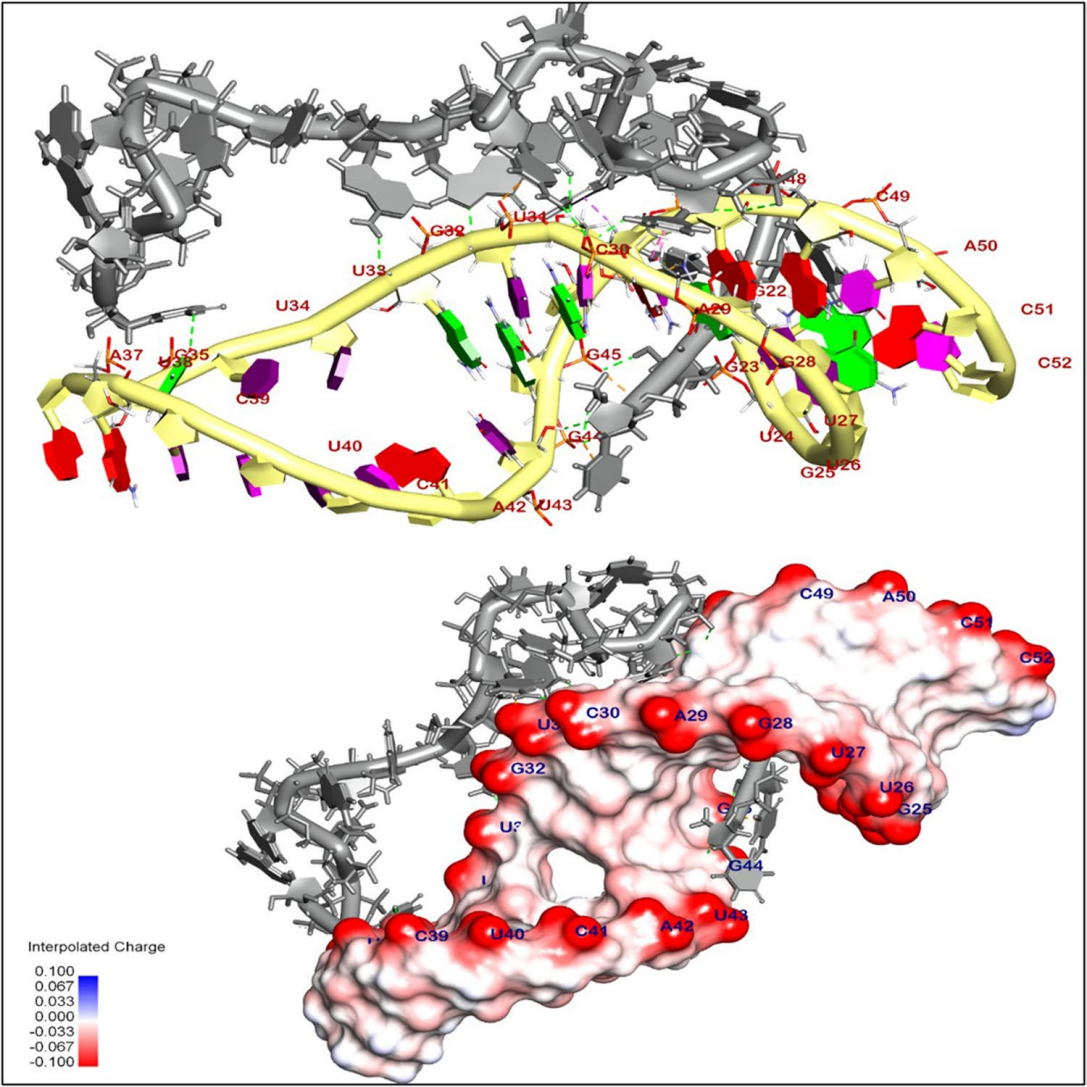
3D orientation and surface mapping of siRNA against oncogenic miRNA-21 precursor target site

The binding mode of interaction of selected siRNA (gray) with miRNA-21(yellow) exhibited an energy binding of − 18.98 kcal/ mol. siRNA was interacted by seventeen hydrogen bonds with Gua22, Gua23, Cyt49, Ade47, Gya45, Gua44, Cyt46, Gua32, U31, Cyt30, and Gua35, additionally formed seven hydrophobic π-interactions with Ade29, Cyt46, Ade47, Ura31, and Gua44 (Fig. 1 and Table 1).

**Table 1:** Displays the (DG, RMSD) values in kcal/mol for the interaction between siRNA and the target site of the oncogenic miRNA-21 precursor

Targets	Inhibitor	Docking (affinity) score (kcal/mol)
Oncogenic miRNA-21 precursor	siRNA	− 18.98

### Molecular docking of EGFR binding

**Fig. 2:**
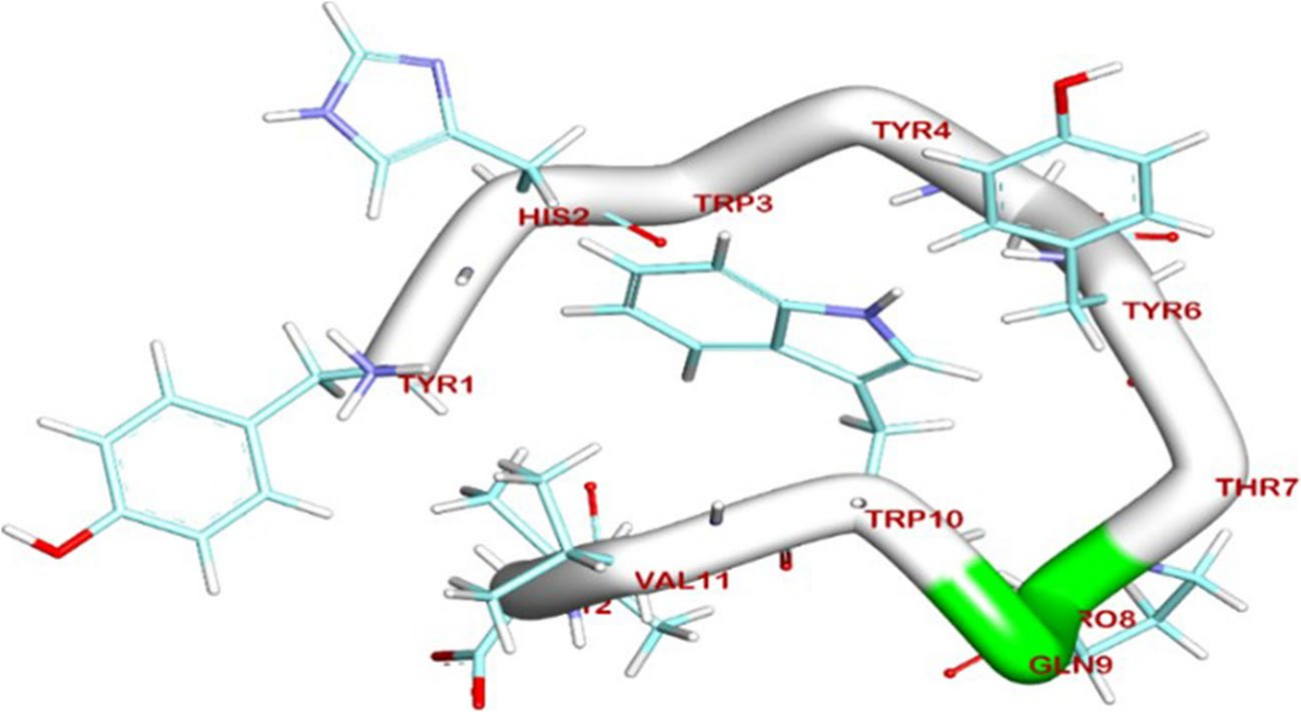
Predicted 3D structure of target peptide

**Fig. 3:**
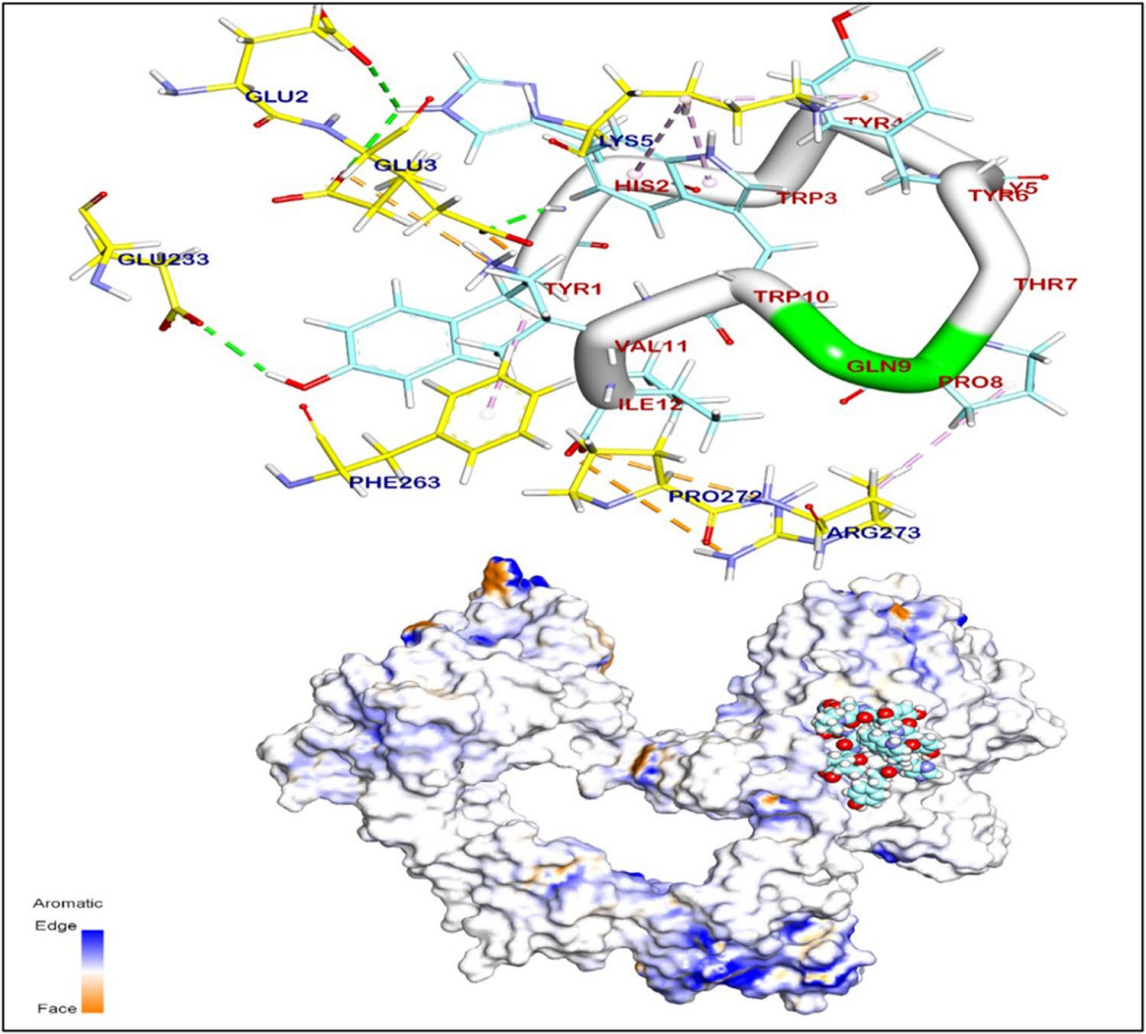
The 3D orientation of peptide interaction with extracellular domain of EGFR TK

Enhancing our understanding of the EGFR protein’s structure and the molecular interactions between ligand and receptor molecules will advance our comprehension of the intracellular transport of receptor-mediated endocytosis, as well as the distribution characteristics of GE11 conjugations both in vitro and in vivo. Herein, the BIOVIA Discovery Studio Visualizer generated the 3D and 2D binding modes. All the data collected from this process can be found in Table 2. The de-novo prediction of 3D structure from the peptide sequence was prepared by the I-TASSER server and visualized by BIOVIA Discovery Studio 2019 software (Fig. 2). Interestingly, the protein-peptides docking results showed that the binding mode of the extracellular domain of EGFR TK (PDB code 3QWQ) (https://www.rcsb.org) with predicted peptides exhibited an energy binding of − 52.62 kcal/mol; the 3D and 2D binding modes were generated by the BIOVIA Discovery Studio Visualizer and all data were collected in Table 3. The extracellular domain of EGFR/peptide complex formed four hydrogen bonds between Glu233, Glu2 and Glu3 against Tyr1and His2, respectively (Fig. 3). On the other hand, Arg273 and Glu3 interacted by ionic interaction with Ile12 and Tyr1. Additionally, peptide interacted with EGFR TK by five hydrophobic π-interactions with Lys5, Phe263, and Arg273.

**Table 2:** Five top final models of tested peptide predicted by I-TASSER

PDB HIT	C-Score
Model 1	− 0.48
Model 2	− 2.75
Model 3	− 5.00
Model 4	− 4.62
Model 5	− 5.00

C-score is typically in the range of [− 5, 2], where a C-score of a higher value signifies a model with a higher confidence and vice-versa

**Table 3:**
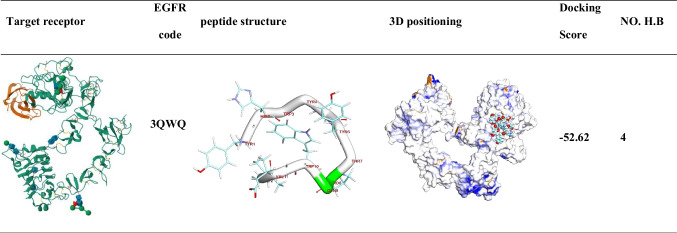
Molecular docking of extracellular domain of EGFR TK (PDB code 3QWQ) with predicted peptide as inhibitor

### Characterization of GE11-siRNA-CSNPs

**Fig. 4:**
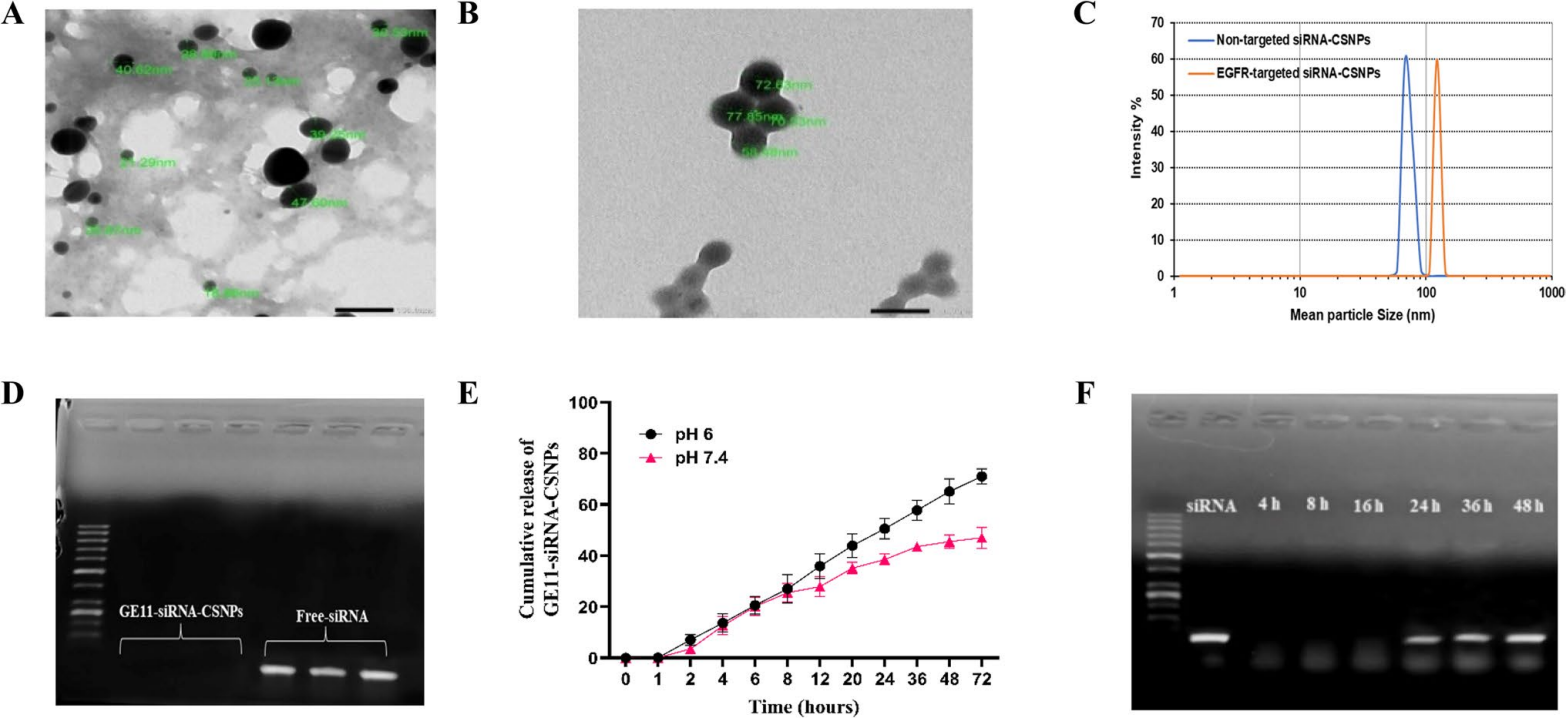
Physicochemical characteristics of GE11-siRNA-CSNPs. **A** TEM results of non-targeted siRNA-CSNPs, **B** TEM results of GE11-siRNA-CSNPs, **C** DLS of non-targeted siRNA-CSNPs and GE11-siRNA-CSNPs, **D** binding efficiency of GE11-siRNA-CSNPs, **E** cumulative release of GE11-siRNA-CSNPs, and **F** gel electrophoresis was used to examine the serum stability of GE11-siRNA-CSNPs

As shown in Table 4, the obtained data from the zeta sizer showed that the size of the siRNA-CSNPs increases noticeably when EGFR-peptide was added to the polymer from 68.10 to 122.00 nm. Moreover, polydispersity index (PDI) values of GE11-siRNA-CSNPs and non-targeted siRNA-CSNPs range from 0.2 to 0.4 also representing a stable system. The morphologies of several siRNA-CSNPs and GE11-siRNA-CSNPs formulations were examined using TEM. Images of non-targeted siRNA-CSNPs show a spherical-like shape with tightly packed chitosan surrounding the siRNA (Fig. 4A). However, GE11-siRNA-CSNPs showed spherical shape and larger particle size, confirming the observation made from the DLS measurement (Fig. 4B). For many nanoparticle formulations, the particle size from the TEM analysis is smaller than the size acquired from the DLS measurement (Fig. 4C). The obtained formulations’ siRNA encapsulation efficiency was then evaluated, and it was found to be almost 97%. Too, agarose gel electrophoresis was used to detect siRNAs that were effectively linked to chitosan nanoparticles. Figure 4D shows that the absence of a band provides additional evidence that siRNAs were effectively encapsulated within chitosan nanoparticles. In addition, the pH-responsive release of siRNA from the produced nanoparticles was assessed using a drug release study conducted in two distinct pH mediums (pH 6 and pH 7.4), which correspond to the acidic and physiological pH conditions, respectively. The release of siRNA from GE11-siRNA-CSNPs at pH 7.4 was very sluggish, as seen in Fig. 4E, and after 72 h, the cumulative release was determined to be close to 44%. Conversely, it is evident that the system exhibits a quicker rate of drug release; that is, 73% of siRNA was released in the same amount of time at an acidic condition. Further, with gel electrophoresis, the serum stability of GE11-siRNA-CSNPs was investigated at predetermined intervals. According to the findings, siRNA release began at 24 h, and after 36 h, nanoparticles completely emptied siRNA (Fig. 4F). Based on the current findings, evaluating the interaction between GE11-siRNA-CSNPs and serum proteins should provide significant knowledge for predicting their efficacy in biological systems.

**Table 4:** Particle size, polydispersity Index (PDI), and zeta-potential of GE11-siRNA-CSNPs and non-targeted siRNA-CSNPs

Prepared nanoparticles	Particle size (nm)	Polydispersity index (PDI)	Zeta-potential (mV)
Non-targeted siRNA-CSNPs	68.10 ± 0.3	0.434 ± 0.08	+ 35.8 ± 2.7
GE11-siRNA-CSNPs	122.00 ± 0.8	0.290 ± 0.03	+ 27.5 ± 2.3

### EGFR expressions and cytotoxic effects of prepared nanoparticles

**Fig. 5:**
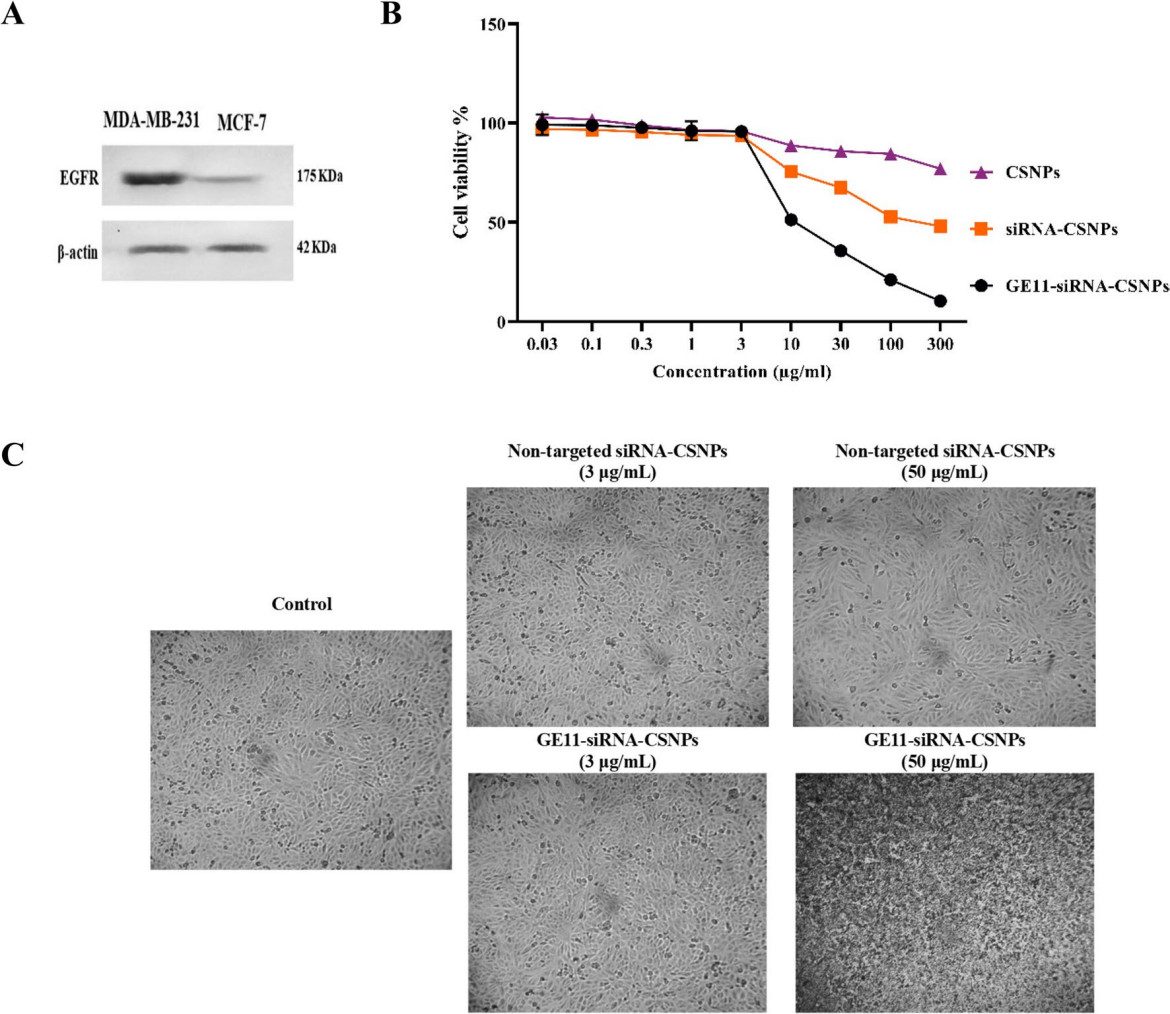
Evaluation of EGFR expression and cytotoxicity of prepared nanoformulations. **A** EGFR expression in TNBC and MCF7 cell lines, **B** cytotoxicity of CSNPs, non-targeted siRNA-CSNPs, GE11-siRNA-CSNPs, and **C** Representative light microscopy imaging of treated MDA-MB-231 cells. Data are shown as mean ± SEM (*n* = 3)

The expression of EGFR in MDA-MB-231 and MCF7 cells was first contrasted. According to the results of the Western blot study, EGFR was expressed most strongly in MDA-MB-231 cells and least in MCF7 cells (Fig. 5A). For the purpose of researching the precise targeting effects of GE11-siRNA-CSNPs, MDA-MB-231 cells with high EGFR expression were chosen. To investigate the biocompatibility of the delivery material in MDA-MB-231 cells, we assessed the cytotoxicity of chitosan both on its own and when loaded with siRNA. Chitosan alone did not appear to be hazardous to cell lines in MTT assays; the IC_50_ was not detectable (ND). The cytotoxic impact of targeted and non-targeted chitosan nanoparticles on MDA-MB-231 cells is then assessed. As expected, the cytotoxic effect of the GE11-siRNA-CSNPs is higher than nontargeted siRNA-CSNPs in the MDA-MB-231 cells because these cells are overexpressing EGFR with IC_50_ 11.83 and 262.04 µg/mL for GE11-siRNA-CSNPs and non-targeted siRNA-CSNPs, respectively (Fig. 5B and Table 5). According to these findings, all siRNA-loaded formulations begin to exhibit lethal effects 48 h after treatment. Most importantly, the absence of toxicity from blank chitosan indicates that the cytotoxicity observed from siRNA-CSNPs is caused by siRNA activity on miRNA-21. Interestingly, the images obtained from the light microscope demonstrated that the number of attached cells decreased as GE11-siRNA-CSNPs concentration increased in MDA-MB-231 cells revealed by a non-significant change in MDA-MB-231 cells treated with same concentrations of non-targeted siRNA-CSNPs (Fig. 5C).

**Table 5:** IC_50_ (µg/mL) of tested compounds against TBNC cells

Prepared nanoparticles	IC_50_
Chitosan nanoparticles (CSNPs)	Non-detectable (ND)
Non-targeted siRNA-CSNPs	262.04 µg/mL
GE11-siRNA-CSNPs	11.89 µg/mL

### Silencing of miRNA21 gene expression, colony formation, and cell migration by GE11-siRNA-CSNPs in TNBC Cells

**Fig. 6:**
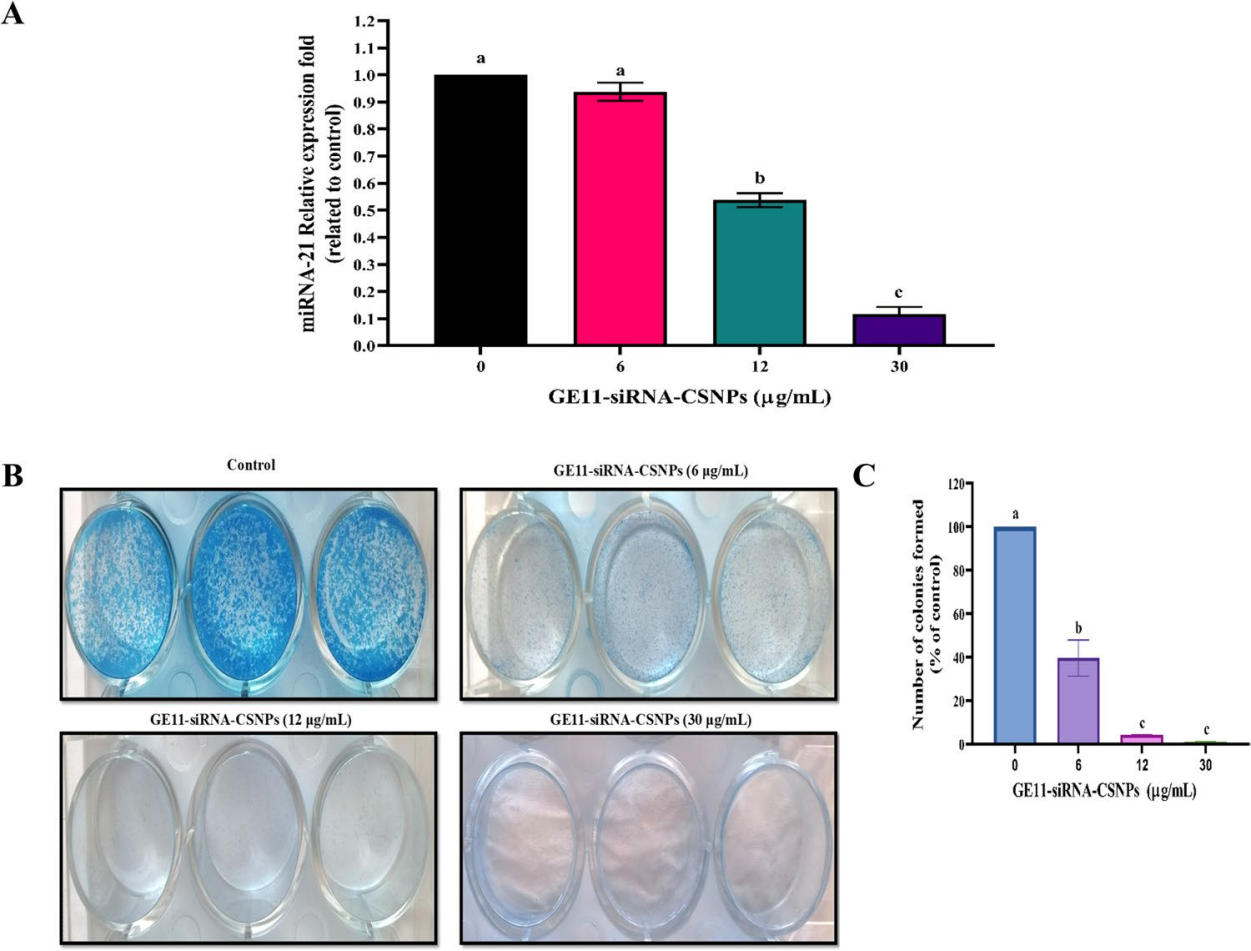

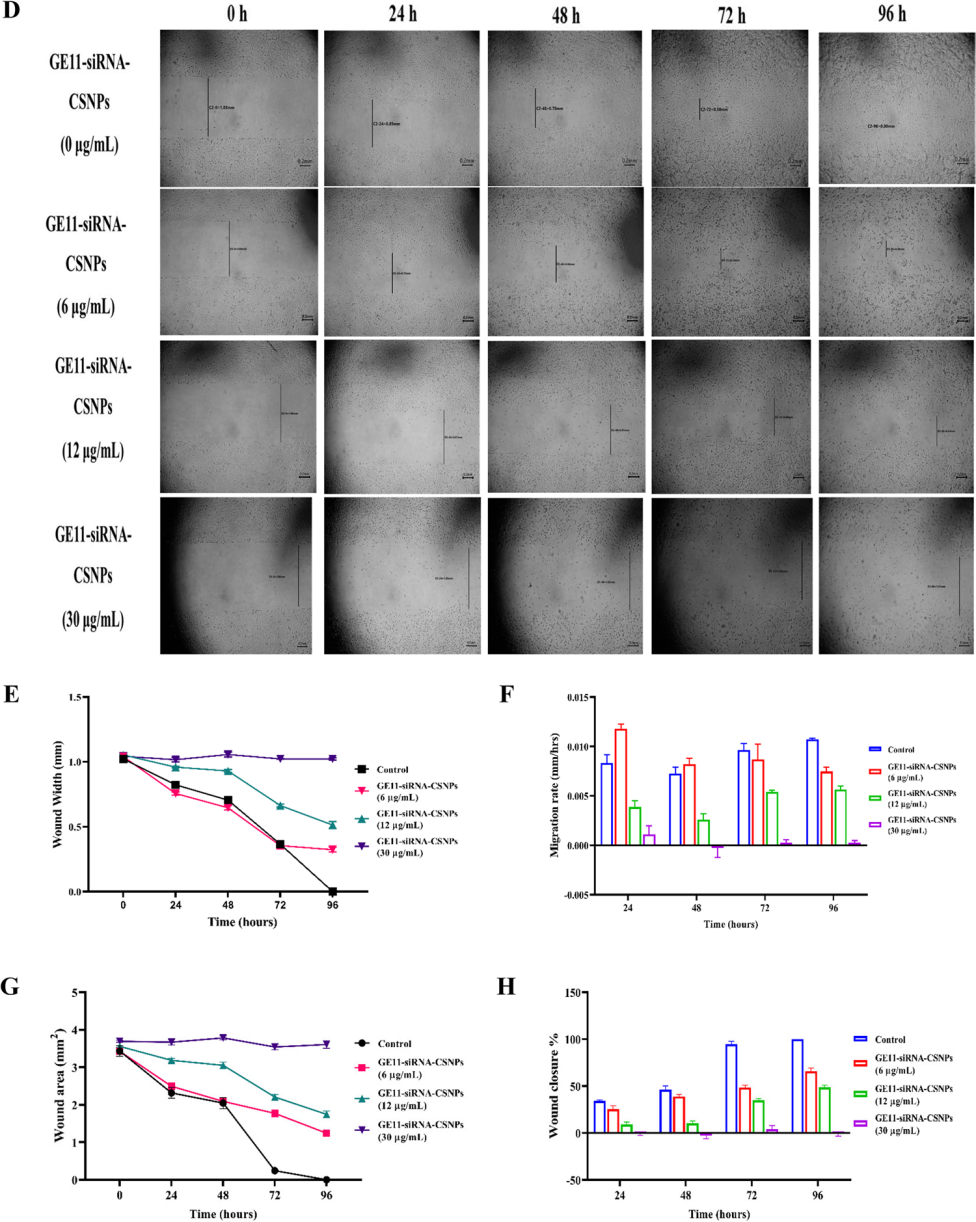
Selective inhibition effects of GE11-siRNA-CSNPs on MDA-MB-231 cells. **A** miRNA-21 expression; data are shown as mean ± SEM (*n* = 3); different letters indicate significant difference. **B** Colony forming assay, **C** quantitative number of colonies, **D** concentration and time-dependent cell migration assay, **E** wound width, **F** migration rate, **G** wound area, and **H** wound closure %

The capacity of current formulation to suppress the expression of the miRNA-21 gene in MDA-MB-231 cells was assessed using quantitative real-time-polymerase chain reaction (qRT-PCR). After treatment with different doses of GE11-siRNA-CSNPs, the amount of miRNA 21-specific mRNA expression was measured. The GE11-siRNA-CSNPs demonstrated a concentration-dependent silencing effect on miRNA-21 expression, as compared to cells that were not treated. miRNA-21 expression shows a marked increase in MDA-MB-231 cells that are not treated with prepared nanoparticles (Fig. 6A). To learn more about how GE11-siRNA-CSNPs affect cancer cell development, the colony formation assay was tested on MDA-MB-231 cells. Figure 6B and C demonstrates that GE11-siRNA-CSNPs significantly decreased colony formation in TNBC cells in a dose-dependent manner, as compared to untreated cells. The MDA-MB-231 cell line exhibits a significant propensity for metastasis in cancer. Cell migration assay was utilized in order to investigate the impact of GE11-siRNA-CSNPs on MDA-MB-231 cell motility. Cells treated with GE11-siRNA-CSNPs (0, 6, 12, and 30 µg/mL) demonstrated that GE11-siRNA-CSNPs hindered wound closure and migration rate at dose and time dependent manner (Fig. 6D). Also, the diameter of the wound exhibited a lower propensity to close as compared to untreated cells at dose and time dependent manner and the bulk of the wound region was filled in the untreated MDA-MB-231 cells while the GE11-siRNA-CSNPs still had a gap suggesting that GE11-siRNA-CSNPs has anti-migration properties on MDA-MB-231 cells (Fig. 6E–H).

### Inhibition of cell cycle and stimulation of ROS production by GE11-siRNA-CSNPs

**Fig. 7:**
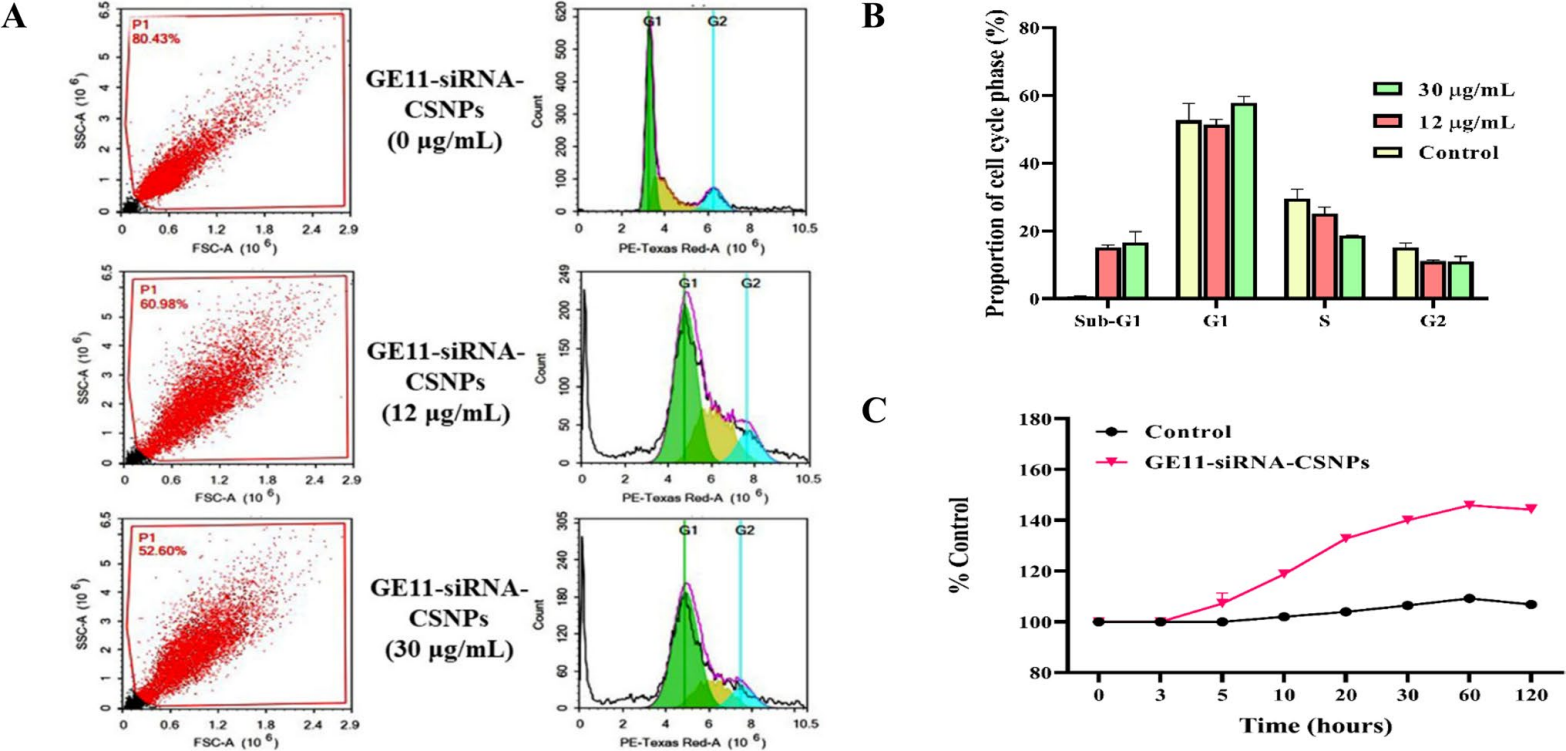
Anticancer effects of GE11-siRNA-CSNPs in MDA-MB-231 cells. **A** Cell cycle analysis by flow cytometry, **B** the proportion of each stage of cell cycle, and **C** ROS production

The mechanism known as the cell cycle consists of four phases: growth (G1), DNA replication (S), preparation for mitosis (G2), and mitosis (M). Anti-mitotic agents stop cells from advancing through the mitotic stages. Consequently, many cells fail to enter the G1 phase of the cell cycle due to their restriction during the initiation step. These cells are in a separate phase known as the sub-G1 phase. Using flow cytometry, the current work examined the effects of GE11-siRNA-CSNPs on MDA-MB-231 cells throughout their cycle at concentrations (0, 12, and 30 µg/mL) (Fig. 7A). The percentages of sub-G1 phase, which represent apoptotic cells, showed a significant increase following treatment with GE11-siRNA-CSNPs (Fig. 7B). The results obtained demonstrate the significant capability of GE11-siRNA-CSNPs to cause cell arrest in the sub-G1 phase. In this study, we measured the ROS level following GE11-siRNA-CSNPs treatment because the inhibition of miRNA-21 is a specific anticancer strategy that could cause cancer cells to apoptosis in a ROS-dependent manner. Figure 7C demonstrates that after 5 min of treatment, GE11-siRNA-CSNPs markedly increase ROS production in MDA-MB-231 cells, with a gradual decline occurring after 60 min. Significant ROS production was caused by GE11-siRNA-CSNPs, which increased anticancer efficacy.

### Initiation of apoptosis and inhibition of EGFR-mediated proliferation cascades by GE11-siRNA-CSNPs in TNBC cells

**Fig. 8:**
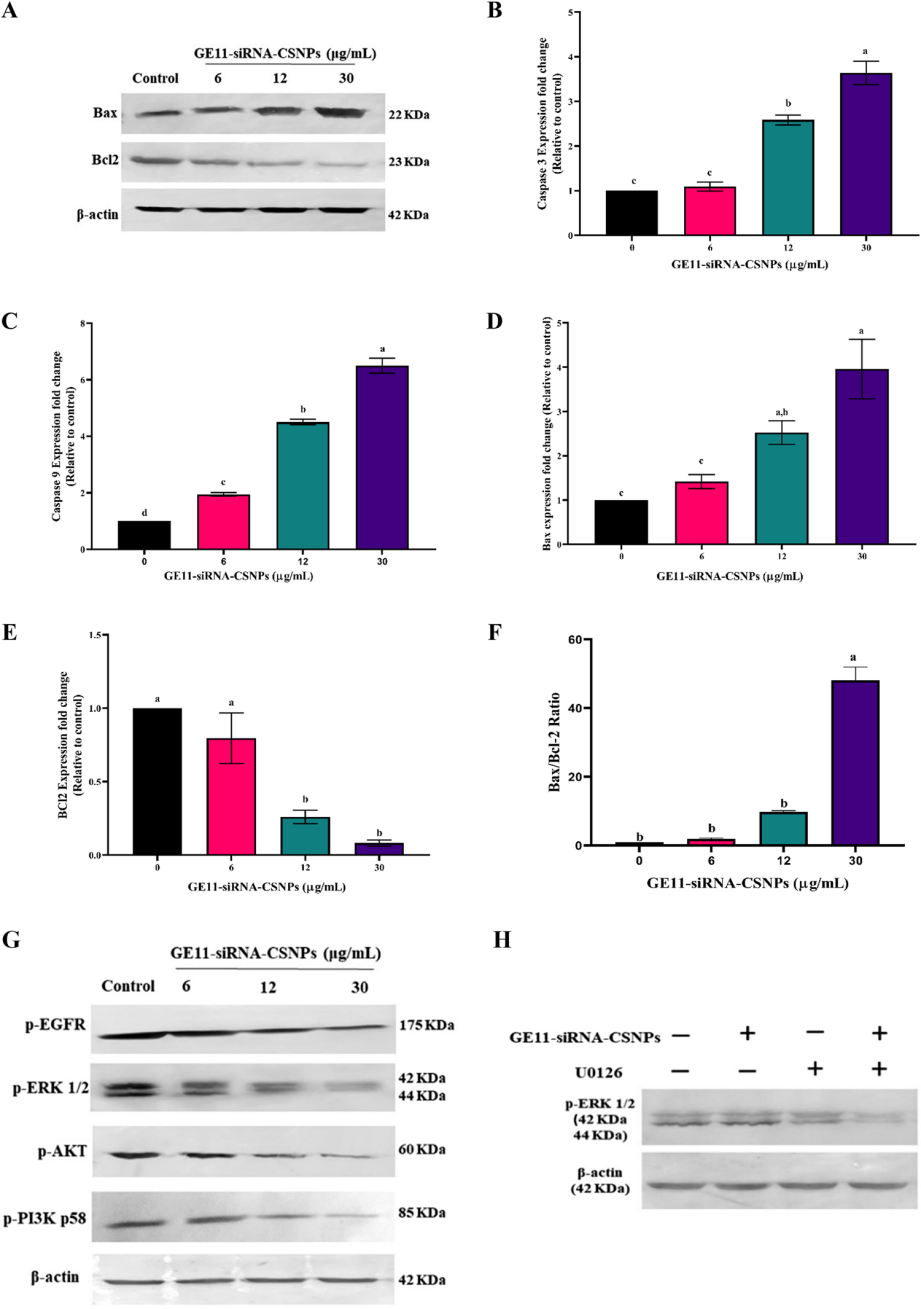
Antiproliferative efficiencies of GE11-siRNA-CSNPs in MDA-MB-231 cells. **A** Immunoblot of Bax, BCL2, and β-actin proteins, **B** gene expression profile of caspase 3, **C** caspase 9, **D** Bax, **E** BCL2, **F** Bax/ BCL2 ratio; values are expressed as mean ± SE, (*n *= 3), means for the same parameter with different letters in each bar are significantly different (*p* < 0.01). **G** immunoblot of p-EGFR, p-AKT, p-PI3K, p-ERK1/2, and β-actin proteins; and **H** MDA-MB-231 cells were treated with GE11-siRNA-CSNPs and/or U0126 for 24 h, and the protein levels of p-ERK1/2 were detected

To examine the process of induced cell death through current formulation, we analyzed the expression of key components related to cell survival, such as BCL2 and Bax, using RT-PCR and immunoblotting techniques. The immunoblot results consistently showed that the GE11-siRNA-CSNPs had an additional impact on enhancing Bax expression and reducing BCL2 expression in MDA-MB-231 cells, in a concentration-dependent manner (Fig. 8A). The current results indicate that the gene expression levels of pre-apoptotic genes; caspase-3, caspase-9, and Bax were significantly increased after treatment with GE11-siRNA-CSNPs in a dose-dependent manner (Fig. 8B–D). However, in GE11-siRNA-CSNPs-treated cells, anti-apoptotic gene, BCL2 showed a markedly reduced gene expression in a dose-dependent manner with a significant increase in Bax/BCL2 ratio (Fig. 8E and F). Using immune blot analysis, we examined EGFR expression and phosphorylation, and we found that GE11-siRNA-CSNPs reduced EGFR phosphorylation significantly. Additionally, we obtained that GE11-siRNA-CSNPs significantly reduced the phosphorylation of PI3K and AKT in a concentration-dependent manner, indicating that they may inhibit the EGFR-mediated PI3K/AKT pathway. The outcomes also showed that the GE11-siRNA-CSNPs downregulated ERK1/2 phosphorylation in a dose-dependent manner in TNBC cells. These results suggested that by inhibiting the EGFR-mediated ERK cascade, GE11-siRNA-CSNPs caused cell death in MDA-MB-231 cells (Fig. 8G). Importantly, U0126, an ERK selective inhibitor, was utilized in order to demonstrate and verify the decrease in p-ERK1/2 protein levels by inhibition of miRNA-21 expression. We treated the cells with GE11-siRNA-CSNPs or/and U1026 for a period of 24 h and then assessed the p-ERK1/2 expression. As can be seen in Fig. 8H, ERK1/2 phosphorylation was greatly inhibited when TNBC cells were treated with a combination therapy consisting of GE11-siRNA-CSNPs and/or U0126, with the highest suppression was seen in MDA-MB-231 cells treated with the combination therapy. According to the current data, the ability of miRNA-21 to enhance phosphorylation of ERK1/2 was no longer observed after treatment with GE11-siRNA-CSNPs.

### Molecular docking and binding mode of miRNA-21 with AKT

**Fig. 9:**
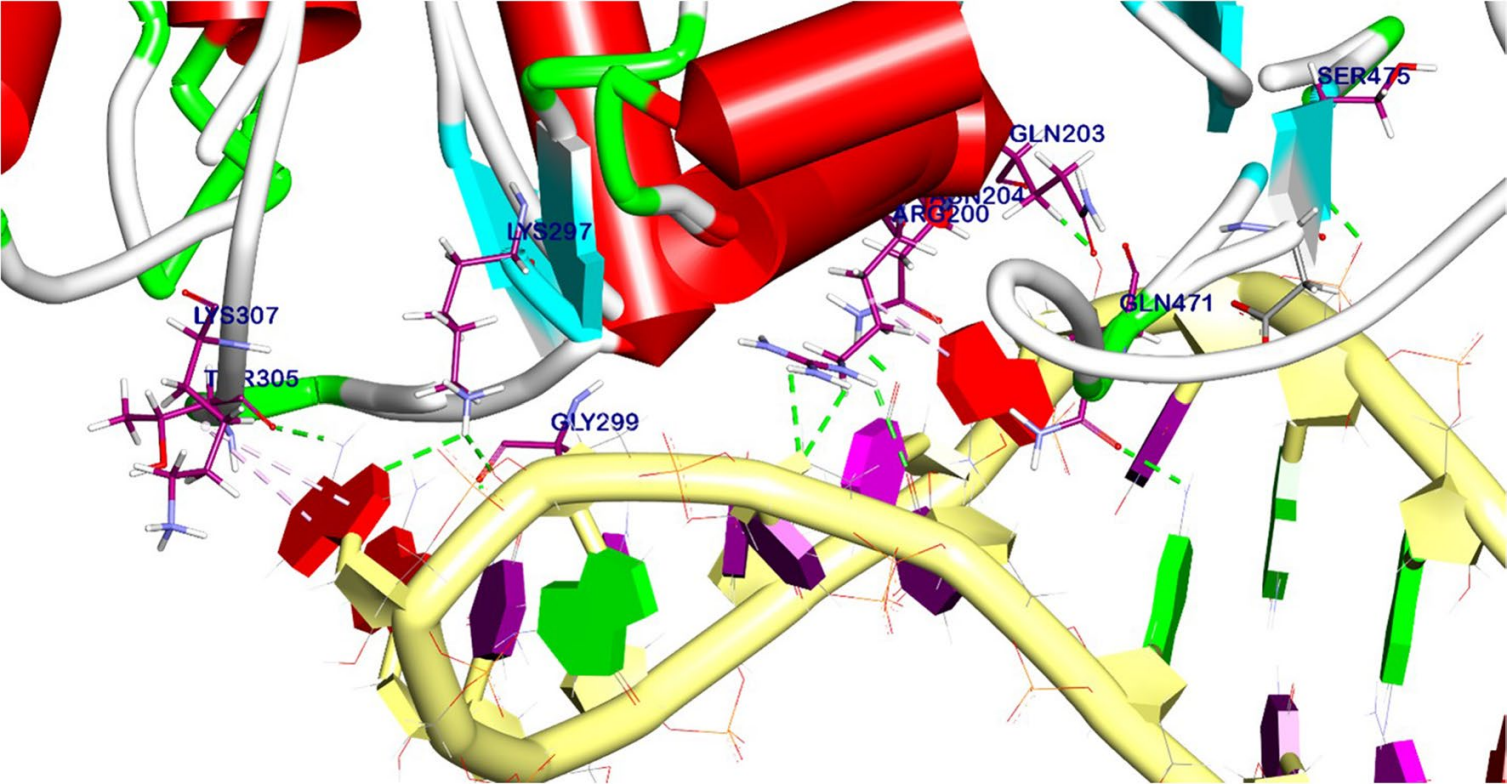
3D binding mode between miRNA-21 and AKT

An in-silico analysis was conducted to evaluate the impact of miRNA-21 on the AKT pathway, which regulates tumor cell growth and apoptosis. The results showed that the binding mode of miRNA-21 with AKT exhibited a binding energy of − 67.58 kcal/ mol; it was observed that miRNA-21 has a high affinity to AKT by interacting with essential domains by three hydrophobic π-interactions with Tyr305 and Arg200, additionally formed eleven hydrogen bonds with Lys307, Lys297, Gly299, Arg200, Asn204, Gln203, Ser475, and Gln47 (Fig. 9). The 3D and 2D binding modes were generated by the BIOVIA Discovery Studio Visualizer and all data were collected in Table 6.

**Table 6:**
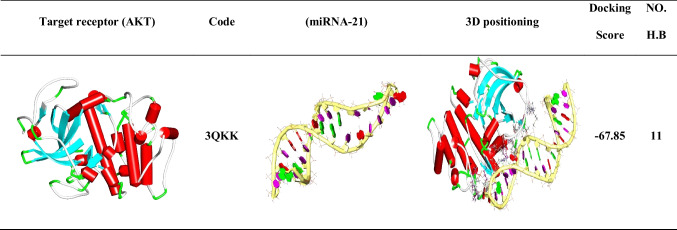
Molecular docking of interaction between miRNA-21 with AKT target site

### Hypoxic cytotoxicity assay

**Fig. 10:**
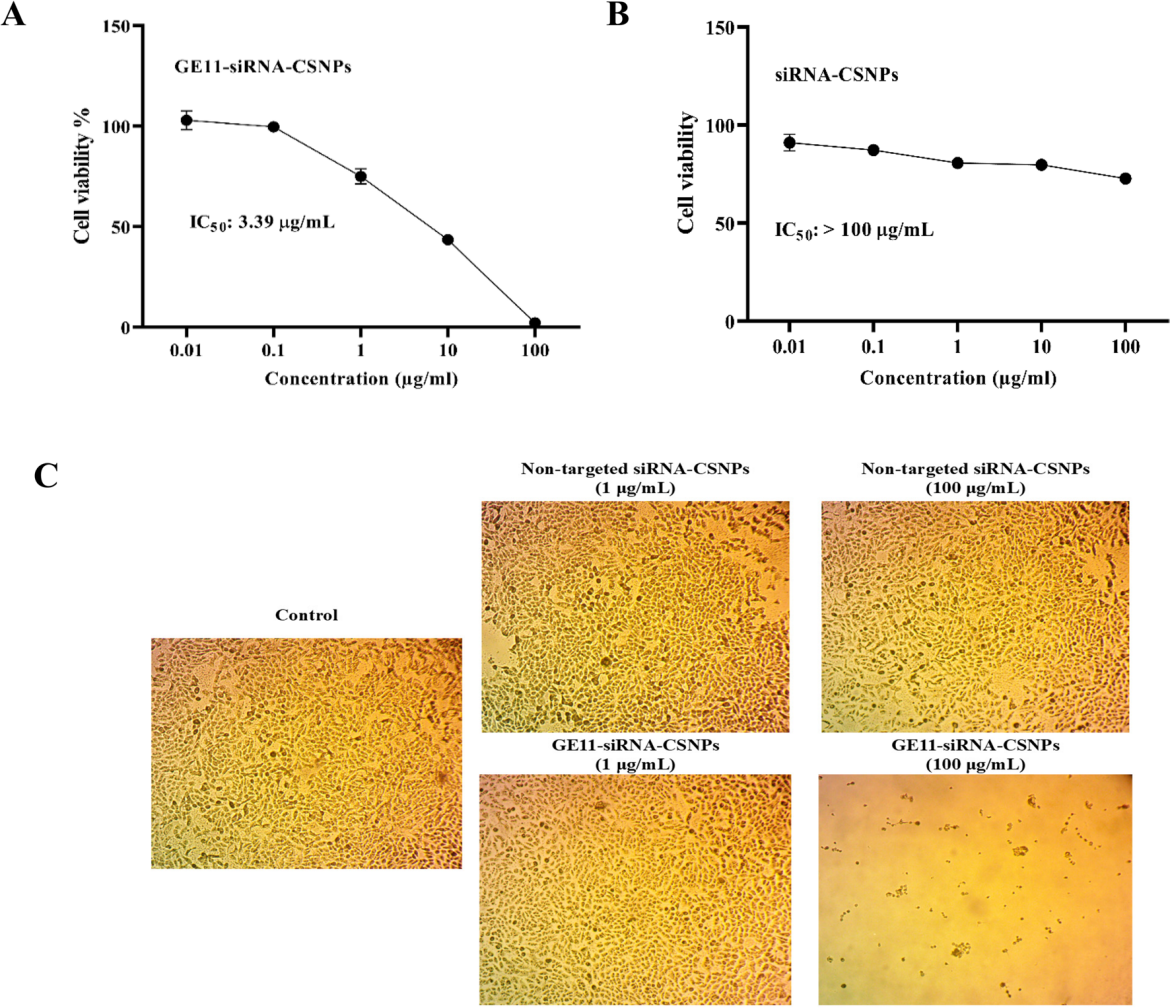
Assessment of hypoxic cytotoxicity of GE11-siRNA-CSNPs. **A** Hypoxic cytotoxicity of GE11-siRNA-CSNPs, **B** hypoxic cytotoxicity of non-targeted siRNA-CSNPs, and **C** representative light microscopy imaging of treated MDA-MB-231 cells. Data are shown as mean ± SEM (*n* = 3)

The hypoxic cytotoxicity test simulates the cancer microenvironment to assess the anticancer properties of prepared nanoformulations. The metabolism of cancer cells is changed, and the majority of solid tumors have far lower oxygen concentrations than normal tissues. Because these hypoxic environments alter how cancer cells react to medications, evaluating a drug’s anticancer effects in these settings will accurately represent the cytotoxic effect and more closely mimic the cancer microenvironment. The current results demonstrated that GE11-siRNA-CSNPs have a greater cytotoxic effect compared to the nontargeted siRNA-CSNPs in the MDA-MB-231 cells, with IC_50_ value of 3.39 µg/mL for GE11-siRNA-CSNPs (Fig. 10A–C).

## Discussion

Multiple mechanisms of resistance to chemotherapy drugs and potential solutions to address this problem with siRNA delivered by nanocarriers have been documented in the literature. siRNA serves as an effective method over small molecule therapies to inhibit the overexpression of several oncogenes. However, properly introducing siRNA into tumor cells intracellularly and blocking the target gene is a challenging task. The development of nanoparticles with a large surface area and small size has allowed for the targeted delivery of siRNA therapies (Lee et al. 2023). The concept of creating nanoscale bioactive that efficiently aggregate in breast cancer cells while exhibiting lower toxicity was made possible by nanotechnology (Bora et al. 2012). Furthermore, because nanoparticles may efficiently cross and accumulate within tumor cells, they have several potential benefits. These have extended systemic retention following surface alteration and are easily tracked to assess the progress in vivo. Additionally, these lessen the negative effects of the bio-actives while also increasing therapeutic response.

Attractively, targeting the tumor microenvironment’s contributing elements can significantly slow the course of cancer. Even though research has already demonstrated the significance of miRNA-21 as an oncogene that regulate tumor formation and progression in a range of tumor types, more investigation is still required to fully understand its novel target genes, exact molecular mechanisms, and therapeutic potential (Lopez-Santillan et al. 2018). For passive tumor targeting, we have created a functionalized siRNA delivery approach, GE11-siRNA-CSNPs, for targeting and inhibiting miRNA-21 expression as a method to effectively cause death in TNBC cells that overexpress the EGFR. One such surface receptor that is frequently seen overexpressed on the surface of malignant tumors is EGFR. In order to subsequently bind the EGFR-binding peptide, we derivatized the chitosan backbone with heterobifunctional PEG, which preserved the particle’s stability profile and increased its surface positive charge, enabling it to bind the specific GE11 peptide. Chitosan nanoparticles have gained widespread acceptance as a promising therapeutic carrier (Jadidi-Niaragh et al. 2016). When anticancer medications are added to chitosan nanocarriers, side effects are decreased and the therapeutic benefit of the pharmaceuticals in the treatment of solid tumors is increased (Vivek et al. 2013). Avoiding the use of the EGFR antibody was necessary since it is larger in size than the EGFR-binding peptide. This size difference often leads to steric resistance when trying to attach it to the surface of the nanoparticles, besides the antibody has limited ability to spread through tissues (Proske et al. 2005). In this instance, PEG, siRNA, and EGFR-targeting peptide surface-modified CSNPs were utilized. PEG can increase the amount of time that nanoparticles are in the bloodstream, whereas GE11 peptide improves cellular uptake and siRNA inhibits miRNA-21 over expression in TNBC cells.

In the current work, siRNAs against miRNA-21 were loaded onto blank and GE11-siRNA-CSNPs, and their size and shape were examined. According to the size analysis of these formulations, siRNA-CSNPs produces particles with an appropriate size and comparable with TEM results. According to prior research, nanoparticles with a diameter of 10 nm or less can be flushed out of the body by the kidneys, whereas those with a diameter of 300 nm or more can be removed from the bloodstream by the reticuloendothelial system (Fox et al. 2009; Kobayashi et al. 2013). In line with previous reports, the lower the value of the PDI, the higher the possibility of finding a monodisperse system (Rao et al. 2011; Mudalige et al. 2019). The capacity to bind and encapsulate siRNA is unaffected; however, the net positive charge of the chitosan may have changed, causing a reduction in particle packing density. This was verified by measuring the zeta-potential for the GE11-siRNA-CSNPs, which dropped in the targeted nanoparticles. This suggests a net decrease in positive charge, which would affect its interaction with negatively charged siRNA and result in a larger size for the targeted nanoparticles, suggested that chitosan is typically utilized as a reliable and effective vector (Ragelle et al. 2013). Also, the EGFR peptide’s inclusion resulted in a drop in the values of PDI for the produced chitosan nanoparticles. The siRNA encapsulation effectiveness of the resulting formulations was subsequently assessed and determined to be approximately 97%. This finding demonstrated that while the presence of the peptide influences the nanoparticle’s assembly, it has no bearing on the efficiency of encapsulation, and peptide-modified chitosan may still effectively encapsulate siRNA in spite of charge compensation (Ragelle et al. 2014). Too, the results showed that siRNA could be released in an acid-triggered way by the produced GE11-siRNA-CSNPs. Since the pH of tumors is lower than that of healthy tissues and their microenvironments are acidic, the pH-dependent drug release method of GE11-siRNA-CSNPs may be useful in causing GE11-siRNA-CSNPs to accumulate in tumor cells. Furthermore, nuclease resistance is essential for the effective transport of siRNA, both in vitro and particularly in vivo. Herein, the interaction between GE11-siRNA-CSNPs and serum proteins can offer valuable insights for predicting their effectiveness in biological systems.

It is worth noting that an in-silico assessment reveals a distinct binding interaction of miRNA-21 with siRNA as well as between extracellular domain of EGFR with synthesized peptides. The in vitro experiments demonstrated that GE11-siRNA-CSNPs induced significant cytotoxicity in MDA-MB-231 cells after 48 h of incubation, in contrast to the non-toxic effects observed with chitosan alone. These findings demonstrate that chitosan is very biocompatible, and that any cytotoxicity impact caused by the synthesized nanoparticles is attributable to the contribution of siRNA. Over and above, these results revealed that inhibition of the function of miRNA-21 lessened the viability of TNBC cells. High silencing activity of siRNA at the gene level demonstrated that the chitosan delivery vector is capable of both protecting the payload from degradation and releasing it in time for in vitro activity. It also reduced the tendency of TNBC cells to heal, in contrast to untreated cells (Egorova et al. 2021; Yang et al. 2023a, b). Even though breast cancer cells have adapted to higher than usual ROS levels, if intracellular ROS are not removed quickly, the buildup of ROS can still cause tumor cells to undergo apoptosis. And the correlation between the build-up of ROS and the degradation of cells has been demonstrated previously (Cordani et al. 2020). While the membrane transporters facilitate the removal of drugs and ROS, hence playing a role in the development of resistance to several drugs in many forms of cancer (Lee et al. 2019; Nedeljković and Damjanović 2019). More specifically, it has been observed that overexpression of miRNA-21 is linked to the development of multi drug resistance in breast cancer and that miRNA-21 itself causes drug resistance (Najjary et al. 2020). In TNBC, the P-glycoprotein transporter can facilitate the development of resistance to doxorubicin (Fathy Abd-Ellatef et al. 2020). This impact was discovered to be secondary to a decrease in intracellular ROS buildup. These findings have prompted the development of strategies to raise intracellular ROS concentration or disrupt cellular redox equilibrium for cancer treatment (Zou et al.^2017^). It is important to mention that GE11-siRNA-CSNPs induced significant amounts of ROS in a time-dependent manner. This suggests that GE11-siRNA-CSNPs formulation work to further increase intracellular ROS levels in TNBC cells, which in turn leads to increased anticancer activity and apoptosis.

One of the most significant changes in the development of cancer cells is the control of the cell cycle. The fundamental properties of cyclins, checkpoints, and cyclin-dependent kinases change at different stages of the cell cycle and control the development of the cell cycle. The cell cycle may be halted at stages by cellular damage and stress signals, which would make the checkpoints faulty (Papagiannakopoulos et al. 2008). GE11-siRNA-CSNPs were able to induce tumor-initiating cells of triple-negative breast cancer cells to undergo apoptosis and to be arrested in the sub-G1 phase. It has been shown that miR-21 may reduce TP53, a significant tumor suppressor gene implicated in the DNA damage response, which triggers cell cycle arrest and apoptosis (Natarajan 2016). Furthermore, miRNA-21 has been linked to the development of fluorouracil-resistant cells in colorectal cancer that prevents G2/M arrest and inhibits apoptosis (Villunger et al. 1997). The observed outcomes exhibited an increase in the sub-G1 phase that coincided with the assured decrease in the G2/M population, indicating that cells were unable to repair DNA damage and ultimately entered the apoptotic or necrotic phase. According to the current survey, GE11-siRNA-CSNPs have a strong potential to cause cell arrest in the sub-G1 phase.

In most cases, escape from apoptosis is one of the most significant alterations that can occur in cellular physiology, and this might contribute to the expansion of tumors. miRNAs’ small size and capacity to regulate cancer cell development contribute to apoptotic arrest (Nedaeinia et al. 2017; Passos et al. 2023). Numerous anti- and pro-apoptotic proteins are involved in the intricate process of apoptosis. Cancer chemoresistance has been associated with overexpression of anti-apoptotic BCL2 family members (Ding et al. 2020), whereas high amounts of pro-apoptotic proteins, such as caspase 3, Bax, and p53, accelerate apoptosis and make tumor cells more responsive to anticancer therapy (Kim et al. 2007). Many anticancer drugs increase the expression of caspase 3, Bax, and p53, which kills cancer cells (LeBlanc et al. 2002). In this instance, we were able to trigger apoptosis in cancer cells by upregulating Bax and caspases and downregulating BCL2 expression (Scully et al. 2023). The consistent changes in the expression of Bax and BCL2 revealed that the Bax/BCL2 signaling pathway might be a common mechanism to inhibit tumor growth, which is a viable direction for creating methods for treating various malignancies. GE11-siRNA-CSNPs-induced programmed cell death is thought to be mediated by the mitochondria, as evidenced by an elevation in the Bax/BCL2 ratio. Given the considerable increase in apoptotic markers in this study, GE11-siRNA-CSNPs could be employed in combination with other therapeutic agents to treat TNBC. Even if the tumor is resistant to standard therapies, it can be used to sensitize it to other therapeutic agents. Furthermore, by using a natural apoptosis mechanism that is compatible with cell biology, it can be employed as a potential targeted therapy.

In breast tumor tissues, signal transduction has been linked to a number of carcinogenic genes, some of which are suitable targets for cancer treatments. AKT and mTOR, two downstream targets of PI3K signaling, are essential for cell survival, proliferation, and differentiation (Zhang et al. 2017). Activating the PI3K/AKT/mTOR signaling pathways increases the growth of tumor cells. The tumor suppressor PTEN targets AKT to control biological processes such as cell development, differentiation, proliferation, and migration. It is a negative regulator of the PI3K/PTEN/AKT signaling pathways (Hu et al. 2017). miRNAs have the potential to control AKT via its positive or negative effects. These miRNAs stimulated TNBC cells migration and proliferation while also increasing the activity of the PI3K/AKT pathway. Moreover, EGFR overexpression and phosphorylation, which are common mechanisms in epithelial malignancies, are associated with metastasis, chemotherapy resistance, and poor prognosis, making it a promising target for cancer therapy (Nicholson et al. 2001). And the affinity of binding between GE11 and EGFR is directly linked to GE11-modified nanomedicine through EGFR-mediated endocytosis that confirmed by docking analysis. In addition, in miR-21-knockout cells, PTEN is markedly increased, suggesting that miR-21 downregulates PTEN and lessens its inhibitory effect on the PI3K/PTEN/AKT signaling pathway (Chen et al. 2015). Given the promising effect of miRNA in modulating cell proliferation, we investigated whether inhibition of miRNA-21 expression by GE11-siRNA-CSNPs correlates with targeting cell proliferation pathways. Additionally, in tumor tissues, GE11-modified nanoparticles demonstrated a superior targeted aggregation effect and a higher affinity with EGFR. So, we examined EGFR expression, and we discovered that GE11-siRNA-CSNPs reduced EGFR phosphorylation significantly in a concentration-dependent manner. Too, the activation of the EGFR-downstream PI3K/AKT signaling axis is closely related to the protection of cells against apoptosis. Previous research showed that miRNA-21’s regulation might be adjusted to increase cancer cell apoptosis by modulating many target genes involved in cell apoptosis, such as BCL2 and PTEN (Huang et al. 2015). Using immune blot analysis, we found that GE11-siRNA-CSNPs significantly reduced the phosphorylation of PI3K and AKT, indicating that they may inhibit the EGFR-mediated PI3K/AKT pathway.

Numerous investigations have explored the interplay of miRNA and genes. Herein, our research was primarily motivated by the theory that miRNA-21 can interact with proteins like AKT, which promote the growth of TNBC cells. Surprisingly, we discovered that miRNA-21 and AKT, which controls cell proliferation, had a substantial binding affinity of − 67.58 kcal/mol and a favorable possibility of interactions. It was observed that miRNA-21 interacts with essential domains through three hydrophobic π-interactions with Tyr305 and Arg200 with a total of eleven hydrogen bonds formed. These interactions highlight the strong binding between miRNA-21 and AKT. So, targeting and silencing of miRNA-21 inhibits the PI3K/AKT axis to promote cell death and increase drug sensitivity in breast carcinoma cells. This could be the cause of the observed rise in apoptosis in TNBC cells. Additionally, the traditional EGFR downstream Ras/Raf/MEK/ERK pathway is a significant cell proliferation signaling pathway in addition to the PI3K/AKT pathway (Bartholomeusz et al. 2012; El Guerrab et al. 2020). The outcomes showed that the GE11-siRNA-CSNPs downregulated ERK1/2 phosphorylation in a dose-dependent manner in TNBC cells. These results suggested that by inhibiting the EGFR-mediated ERK cascade, GE11-siRNA-CSNPs caused cell death in MDA-MB-231 cells. miRNA-21 has been shown to function as an oncogene in cancer by influencing several pathways that are crucial in the growth of tumors (Wen et al. 2017). It has been demonstrated that the MEK/ERK signaling pathways are regulated by miRNA-21 (Wang et al. 2019). We verified that GE1-siRNA-CSNPs, which specifically target miRNA-21, can prevent the effects of miRNA-21 on the proliferation of TNBC cells via controlling EGFR and subsequently inhibiting the PI3K/AKT and ERK1/2 signaling axis. This was proven by molecular docking and in vitro studies.

Interestingly, elevated concentrations of miRNA-21 can indirectly induce resistance to chemotherapy by facilitating hypoxia, a state that aids tumors in evading the immune system, fostering resistance to chemotherapy, and activating many processes that impact tumor formation. Hypoxia induces the activation of hypoxia-inducible factor HIF-1α, which subsequently promotes the synthesis of miRNA-21 (Hashemi et al. 2023). Cancer cells are able to avoid the reduction in cell division caused by hypoxia by escaping apoptosis. Hypoxic environments modify cancer cell responses to medications, hence studying anticancer drug effects in hypoxic conditions will better imitate the cancer microenvironment and reflect the real lethal effect. An investigation carried out on TNBC cells revealed that GE11-siRNA-CSNPs displayed a potent cytotoxicity effect under hypoxic condition on TNBC cells compared to non-treated cells. Our results suggested that the coupling of siRNA and GE11 with PEG-CSNPs is a potential nanocarrier that enhances the anti-tumor efficacy against TNBC cells. Future research can take into consideration the efficacy of this nanocarrier in cancer therapy for human tumors alone or as an add-on to standard medications.

## Conclusions

A useful strategy to combat and target cancer cells is siRNA treatment instead of using current chemotherapeutic methods (the “druggable strategy”). Molecular docking studies revealed that hydrogen bonds interactions were key factors involved in siRNA and miRNA-21 binding as well as the protein-peptides binding of GE11 peptide and extracellular domain of EGFR TK. The in vitro results revealed that miRNA-21 suppression efficiently suppresses TNBC proliferation and augments apoptosis. GE11-siRNA-CSNPs have been found to increase cancer cell death in different ways; by inhibiting cell proliferation, increasing ROS production, activating mitochondrial-dependent pathways, and inhibiting cell cycle. We verified that GE1-siRNA-CSNPs, a specific target for miRNA-21, can counteract the effects of miRNA-21 on the proliferation of TNBC cells via inhibiting the PI3K/AKT and ERK1/2 signaling axis. Finally, our study suggests that adding GE11-siRNA-CSNPs to typical oncology regimens may enhance cancer treatment. In addition, siRNA may boost breast cancer treatment by overcoming medication resistance. Hence, it is imperative to conduct in vivo studies to assess the clinical applications of GE11-siRNA-CSNPs as an anticancer drug.

## Data Availability

All data generated or analyzed during this study are included in this article.

## Abbreviations

TNBC: Triple-negative breast cancer
EGFR: Epidermal growth factor receptor
MAPK: Mitogen-activated protein kinase
ERK: Extracellular signal-regulated kinases
PI3K: Phosphoinositide 3-kinases
siRNA: Small interfering RNA
CSNPs: Chitosan nanoparticles
PTEN: Phosphatase and tensin homolog
RT-PCR: Real-time PCR
BCL2: B cell leukemia/lymphoma 2 protein
Bax: Bcl-2-associated X protein
Caspases: Cysteine-dependent, aspartate-specific peptidase
PDI: Polydispersity Index
ROS: Reactive oxygen species
